# Adipose Tissue-Derived Stem Cells Have the Ability to Differentiate into Alveolar Epithelial Cells and Ameliorate Lung Injury Caused by Elastase-Induced Emphysema in Mice

**DOI:** 10.1155/2019/5179172

**Published:** 2019-06-10

**Authors:** Eriko Fukui, Soichiro Funaki, Kenji Kimura, Toru Momozane, Atsuomi Kimura, Ryota Chijimatsu, Ryu Kanzaki, Takashi Kanou, Naoko Ose, Masato Minami, Shigeru Miyagawa, Yoshiki Sawa, Meinoshin Okumura, Yasushi Shintani

**Affiliations:** ^1^Department of General Thoracic Surgery, Osaka University Graduate School of Medicine, Suita, Osaka 565-0871, Japan; ^2^Department of Medical Physics and Engineering, Division of Medical Technology and Science, Faculty of Health Science, Osaka University Graduate School of Medicine, Suita, Osaka 565-0871, Japan; ^3^Department of Orthopaedic Surgery, Osaka University Graduate School of Medicine, Suita, Osaka 565-0871, Japan; ^4^Department of Cardiovascular Surgery, Osaka University Graduate School of Medicine, Suita, Osaka 565-0871, Japan

## Abstract

Chronic obstructive pulmonary disease is a leading cause of mortality globally, with no effective therapy yet established. Adipose tissue-derived stem cells (ADSCs) are useful for ameliorating lung injury in animal models. However, whether ADSCs differentiate into functional cells remains uncertain, and no study has reported on the mechanism by which ADSCs improve lung functionality. Thus, in this study, we examined whether ADSCs differentiate into lung alveolar cells and are able to ameliorate lung injury caused by elastase-induced emphysema in model mice. Here, we induced ADSCs to differentiate into type 2 alveolar epithelial cells in vitro. We demonstrated that ADSCs can differentiate into type 2 alveolar epithelial cells in an elastase-induced emphysematous lung and that ADSCs improve pulmonary function of emphysema model mice, as determined with spirometry and ^129^Xe MRI. These data revealed a novel function for ADSCs in promoting repair of the damaged lung by direct differentiation into alveolar epithelial cells.

## 1. Introduction

Mesenchymal stem cells (MSCs) are multipotent stem cells that can be isolated from various tissues [1, 2]. Treatment using MSCs can induce tissue repair by self-renewal, differentiation, and paracrine effects with immunomodulatory properties and anti-inflammatory and angiogenic actions [3]. MSCs are expected to be clinically applicable in regenerative medicine. Adipose tissue-derived stem cells (ADSCs) were first identified as MSCs in adipose tissue in 2001 [4]. ADSCs rapidly proliferate and secrete high amounts of regeneration promoting factors [5]. Recently, the therapeutic utility of ADSCs has been studied in various diseases [6]. Indeed, cellular therapy using ADSCs can induce recovery from injuries such as myocardial infarction [7], cerebral ischemia, and cerebral reperfusion after ischemia [8, 9] and spinal cord injury in animal models [10]. Previous studies reported that ADSCs have the ability to home, immunomodulate, promote repair, and induce direct regeneration of damaged tissue.

Chronic obstructive pulmonary disease (COPD) is a leading cause of mortality throughout the world and is anticipated to become the third leading cause of death by 2020. The influence of exposure to risk factors such as smoking and the aging of the population increase the incidence of COPD [11]. COPD is characterized by an obstructive ventilatory disorder caused by chronic bronchitis and emphysema. Destruction of the alveolar wall causes pulmonary emphysema, which induces overexpansion of the air space and reduces gas exchange capacity. These changes are progressive and irreversible [12]. COPD primarily affects the lungs but may induce significant systemic consequences [13]. However, we currently do not have a definitive treatment for COPD.

ADSCs have been investigated for their utility in respiratory diseases, including models of acute respiratory distress syndrome [14], bronchiolitis obliterans [15], and idiopathic pulmonary fibrosis [16]. Several papers have also shown the effectiveness of ADSCs in COPD. In these studies, ADSCs ameliorate COPD by paracrine effects of several growth factors, such as hepatocyte growth factor (HGF), vascular endothelial growth factor (VEGF), and fibroblast growth factor-2 (FGF-2) [17–21]. These paracrine effects promote the proliferation of alveolar epithelial cells and angiogenesis and inhibit cell apoptosis [22]. Previous investigators have analyzed the histological improvement and paracrine effects, but improvement in respiratory function has not been examined in any detail. Moreover, previous studies have also reported that several types of MSCs, such as bone marrow-derived (BM)-MSCs, amniotic fluid stem cells, and decidua-derived MSCs, differentiate into type 2 alveolar epithelial cells in vitro [9, 23–26]. However, whether ADSCs differentiate into type 2 alveolar epithelial cells is not clear. Furthermore, whether ADSCs differentiate into functional cells (i.e., type 2 alveolar epithelial cells) in vivo and contribute to regeneration is uncertain.

In this study, we investigated whether ADSCs have functional and histological effects in pulmonary emphysema. In addition, we clarified whether ADSCs have the ability to accumulate in the lungs affected by emphysema and differentiate into alveolar epithelial cells.

## 2. Materials and Methods

### 2.1. Isolation and Culture of ADSCs

C57BL/6-Tg(CAG-EGFP) mice were purchased from Japan SLC Inc. (Shizuoka, Japan). ADSCs were isolated from the subcutaneous adipose tissue of 8- to 12-week-old C57BL/6-Tg(CAG-EGFP) mice as described previously [27]. Briefly, we obtained the adipose tissue from the subcutaneous fat pad around the ilium of the mouse and incubated the tissue in phosphate-buffered saline (PBS) containing antibiotic-antimycotic solution (Sigma-Aldrich, St. Louis, MO). We minced the fat pad into fine pieces and then incubated the pieces in Dulbecco's modified Eagle's medium (DMEM) (Sigma-Aldrich) containing 1 mg/ml collagenase II (Sigma-Aldrich) and 1% Penicillin-Streptomycin-Amphotericin B (Sigma-Aldrich) at 37°C for 30 min. The digested tissue was filtered through a sterile 70 *μ*m nylon mesh (BD FALCON, Corning, NY), the filtrate was centrifuged at 1800 rpm for 5 min, and the supernatant was discarded. We resuspended the pellet in a 10 ml culture medium. This process was repeated twice. We seeded ADSCs onto culture dishes with growth medium (DMEM containing 10% fetal bovine serum (FBS) (Sigma-Aldrich) and 1% Penicillin-Streptomycin-Amphotericin B suspension). We dissociated the cells with TrypLE™ Select (Thermo Fisher Scientific, Waltham, MA).

### 2.2. In Vitro Differentiation of ADSCs

For details regarding the protocols for each differentiation, see the Supplemental Experimental Procedures (available here). MLE-12 cells (CRL­2110™) were purchased from the American Type Culture Collection (ATCC, Manassas, VA) and were maintained according to the manufacturer's protocol. MLE-12 cells were mouse lung epithelial cells thus used as a positive control for the analysis of differentiation to alveolar type II cells.

### 2.3. RNA Extraction and Real-Time Quantitative RT-PCR

Total RNA was extracted with an RNeasy Mini Kit (Qiagen, Hilden, Germany). First-Strand cDNA was synthesized from total RNA using PrimeScript RT Master Mix (Takara, Shiga, Japan). We performed quantitative PCR using the THUNDERBIRD Probe qPCR Mix (TOYOBO, Tokyo, Japan) and TaqMan Gene Expression Assay (Applied Biosystems, Foster City, CA) with the CFX96 system (Bio-Rad Laboratories Inc., Hercules, CA). Each sample was assayed in duplicate, and relative expression levels were calculated using the comparative Ct method. The TaqMan Gene Expression Assay identifiers of detected genes were Mm00493214_m1 (EPCAM), Mm01247351_m1 (E-cadherin), Mm00447558_ml (Nkx2-1), Mm00455678_m1 (Sftpb), Mm00488144_m1 (Sftpc), and Mm99999915_g1 (Gapdh).

### 2.4. Immunofluorescence Staining

We fixed the cells with 3.7% formaldehyde in PBS for 15 min at room temperature. The cells were permeabilized in 0.2% Triton X-100/PBS for 15 min at room temperature. After washing three times with PBS, we incubated the cells with blocking solution containing 10% normal goat serum (Vector Laboratories, Burlingame, CA; S-1000) in PBS at room temperature for 30 min. The samples were incubated in primary antibody for 1 h at room temperature. After three washes with PBS, we stained samples with secondary antibody solution for 1 h at room temperature. Nuclei were counterstained with 4′,6-diamidino-2-phenylindole (DAPI) (Vector Laboratories; S-1000).

For the in vivo study, the mice were sacrificed, and the lungs were inflated and fixed by intratracheal instillation of 4% paraformaldehyde at a constant pressure of 25 cmH_2_O. Then, we removed the lungs, embedded them in optimum cutting temperature compound (Sakura Finetek Japan Co. Ltd., Tokyo, Japan), and froze the samples in liquid nitrogen. Sections were cut at 10 *μ*m thick and fixed with 4% paraformaldehyde in PBS for 30 min at room temperature. After washing three times with PBS, we incubated the sections with blocking solution containing 10% normal goat serum in PBS at room temperature for 1 h. The sections were incubated in primary antibody solution overnight at 4°C. After three washes with PBS, sections were stained with secondary antibody solution for 1 h at room temperature. We counterstained the nuclei with DAPI. We stained the samples with the following primary antibodies: rabbit anti-EpCAM antibody (1 : 200; Abcam, Cambridge, UK; ab71916), rabbit anti TTF-1 antibody (1 : 200; Abcam; ab76013), rabbit anti-pro/mature SPB antibody (1 : 100; Abcam; ab40876), and rabbit anti-pro SPC antibody (1 : 100; Abcam; ab170699). Staining was visualized with the following secondary antibodies: Alexa Fluor 488 goat anti-rabbit IgG (1 : 300; Invitrogen, Carlsbad, CA; A11008) and Alexa Fluor 568 goat anti-rabbit IgG (1 : 300; Invitrogen; A11036).

### 2.5. SDS-PAGE and Western Blot Analysis

Cultured cells were treated with the indicated conditions, and proteins were extracted with radio-immunoprecipitation assay buffer (#9806, Cell Signaling Technology Inc., Danvers, MA). Total proteins were separated by sodium dodecyl sulfate polyacrylamide gel electrophoresis (SDS-PAGE) followed by immunoblotting. Western blotting was assessed with the ChemiDoc™ Imaging System (Bio-Rad Laboratories Inc.). The quantification of protein signals was performed with Image Lab software according to the manufacturer's protocol (Bio-Rad Laboratories Inc.). Primary antibodies and their dilutions were as follows: anti-TTF-1 (1 : 2000, Abcam; ab76013), anti-pro+mature SPB (1 : 5000, Abcam; ab40876), polyclonal antibody to SPC (1 : 300, Cloud-Clone Corp., Katy, TX, PAB623Mu02), and purified mouse anti-clathrin heavy chain (1 : 1000, BD Biosciences, Franklin Lakes, NJ, 610500). Secondary antibodies were anti-rabbit IgG and horseradish peroxidase- (HRP-) conjugated antibodies (1 : 2000, Cell Signaling Technology Inc., 7074) and anti-mouse IgG and HRP-conjugated antibodies (1 : 2000, Cell Signaling Technology Inc., 7076). The proteins were visualized with enhanced Western Lightning® Plus-ECL (Perkin Elmer Inc., Waltham, MA, NEL104001EA) according to the manufacturer's recommendations.

### 2.6. Transmission Electron Microscopy

Differentiated ADSCs were fixed with 2.5% glutaraldehyde in 0.1 M phosphate buffer at pH 7.0 for 1 h at room temperature, followed by postfixation with 1% osmium tetroxide in 0.1 M phosphate buffer for 1 h at room temperature. After washing with 0.15 M phosphate buffer, specimens were dehydrated in an ethanol series ranging from 25 to 100% and embedded in Quetol 812 (Nisshin EM, Tokyo, Japan). The samples were cut with a glass knife on an ultramicrotome and poststained with uranyl acetate and lead citrate. Transmission electron microscopic analysis was performed with a Hitachi H-7000 (Hitachi High-Technologies Corp., Tokyo, Japan).

### 2.7. Animal Housing

In this study, animal care complied with the Guide for the Care and Use of Laboratory Animals (National Institutes of Health Publication No. 85-23, revised 1996). All mouse studies were approved by the Ethics Review Committee for Animal Experimentation of Osaka University Graduate School of Medicine. Mice were housed in a standard 12 h light/dark cycle (lights on at 8:00 a.m.) at a constant temperature of 22 ± 1°C with free access to food and water throughout the experiments. To prevent any animal pain, all experimental animals were anesthetized with Medetomidine hydrochloride (ZENOAQ, Fukushima, Japan), Midazolam (Astellas, Tokyo, Japan), and Butorphanol (Meiji Seika Pharma Co. Ltd., Tokyo, Japan).

### 2.8. Experimental Protocol

Emphysema was induced in 8- to 10-week-old C57BL/6J (CLEA Japan, Tokyo) mice by intratracheal administration of PPE (Elastin Products Company Inc., Owensville, MO; 75 IU/kg in 50 *μ*l PBS). Control mice received 50 *μ*l PBS intratracheally. After 3 days, GFP-labeled ADSCs were intravenously administered (5 × 10^5^ cells/mouse in 200 *μ*l saline twice) via a tail vein.

### 2.9. Flow Cytometry and Cell Sorting

Detection of GFP-labeled ADSCs in a mouse lung was performed by FACS on day 7, day 14, and day 21 (*n* = 6 in each group and on each day). To prepare a single-cell suspension from the lungs, the harvested lungs were minced and incubated with DMEM containing 0.7 U/ml Dispase I, 1 mg/ml collagenase type I, 1 mg/ml collagenase type II (Sigma-Aldrich), and 40 mg/ml DNase I (Roche, Basel, Switzerland) for 7 min at 37°C in a shaking water bath. The digested tissue was passed through 70 *μ*m filters to yield single-cell suspensions. Cells were collected by centrifugation (1800 rpm, 3 min). The cells were washed with PBS containing 4% FBS and treated with red blood cell lysis buffer (0.15 M NH_4_Cl, 1 mM KHCO_3_, and 0.1 mM EDTA) for 5 min. We collected the cells with centrifugation (1800 rpm, 3 min) and blocked nonspecific staining with purified rat anti-mouse CD16/CD32 (Mouse BD Fc block) (1 : 50; BD Biosciences; 5543142) for 10 min. Then, we fixed and permeabilized the cells with CytoFix/Cytoperm fixation buffer and permeabilization solution (BD Biosciences; 51-2090KZ) for 20 min. The cells were stained with rabbit anti-pro SPC antibodies (1 : 50; Abcam; ab170699) for 30 min on ice, followed by goat anti-rabbit IgG (APC) (1 : 50; Abcam; ab130805) as the secondary antibody for 30 min on ice. We also used an anti-mouse IgG1 isotype control antibody (APC) (1 : 50; Abcore, Ramona, CA; 1A-632). Data were acquired using a FACS Aria IIIu (BD Biosciences).

### 2.10. Histological Assessment

After the mice were sacrificed, the lungs were inflated and fixed by intratracheal instillation of 4% paraformaldehyde at a constant pressure of 25 cmH_2_O. The lungs were then removed and embedded in paraffin, and 3 *μ*m thick sections of the lung were cut. The sections were stained with hematoxylin and eosin. The alveolar size was evaluated by the mean linear intercept in 10 randomly selected fields for each mouse (*n* = 3) [28, 29].

### 2.11. Micro-CT Imaging

The micro-CT system (R_mCT2, Rigaku Corporation, Tokyo, Japan) was operated at 90 kV and 160 A, and the chest CT was operated in a respiratory reconstruction mode. The scan time was 34 s. Mice were scanned in the supine position with inhalation anesthesia of mixed isoflurane (Pfizer Japan, Tokyo, Japan) and oxygen through a nose cone. The data were analyzed using R_mCT2 software (*n* = 4 in the control group, PPE+/ADSC− group, and PPE+/ADSC+ group on each day).

### 2.12. Pulmonary Function Tests

Assessment of pulmonary function was performed using a Buxco lung function analysis system (BUXCO Electronics, Troy, NY) [30]. After anesthetizing the mouse by intraperitoneal injection of medetomidine hydrochloride, midazolam, and butorphanol, we incised the skin of the neck by about 5 mm. We bluntly dissected the subcutaneous tissues and separated the submaxillary glands into the left and right lobes. When we reached the anterior trachea, we created a small hole in the trachea for cannulation with an 18G tube. We placed the mouse into the chamber and connected the tracheal tube to a port in the ventilator of the respiratory function measuring device. We measured the lung compliance (C chord) and lung vital capacity (*n* = 4-6 per group and on each day) according to the manufacturer's instructions and protocol.

### 2.13. Hyperpolarized ^129^Xe MRI

All MRI measurements were performed with an Agilent Unity INOVA 400WB (Agilent Technologies Inc., Santa Clara, CA) as previously described [31]. Briefly, ^129^Xe atoms were polarized by rubidium- (Rb-) ^129^Xe spin exchange optical pumping using a homemade continuous flow ^129^Xe polarizer. 70% Xe gas (70% Xe, 30% N_2_) was polarized at 0.15 atm, and the hyperpolarized gas was compressed to atmospheric pressure using a diaphragm pump (LABOPORT N86KV.18; KNF Neuberger Gmbh, Freiburg, Germany). After anesthesia, a mask was attached to the mouse, and the mouse inhaled the hyperpolarized ^129^Xe and oxygen by spontaneous breathing. Fractional ventilation (*r*_*a*_) and gas exchange efficiency (*f*_*D*_) of hyperpolarized ^129^Xe MRI were evaluated as previously described [32]. We measured the gas distribution in the lung, alveolar size, and diffusion rate with MRI (*n* = 3-5 per experimental group and on each day).

### 2.14. Statistical Analysis

We obtained all the results from at least three independent experiments. Values were expressed as the mean ± standard deviation (SD). We used the Wilcoxon test to assess the significance between the two groups. The statistical significance of differences among three or more groups was assessed using the Kruskal-Wallis test. A value of *P* < 0.05 was considered to be statistically significant.

## 3. Results

### 3.1. Characterization of ADSCs and In Vitro Differentiation Assay

**Figure 1:**
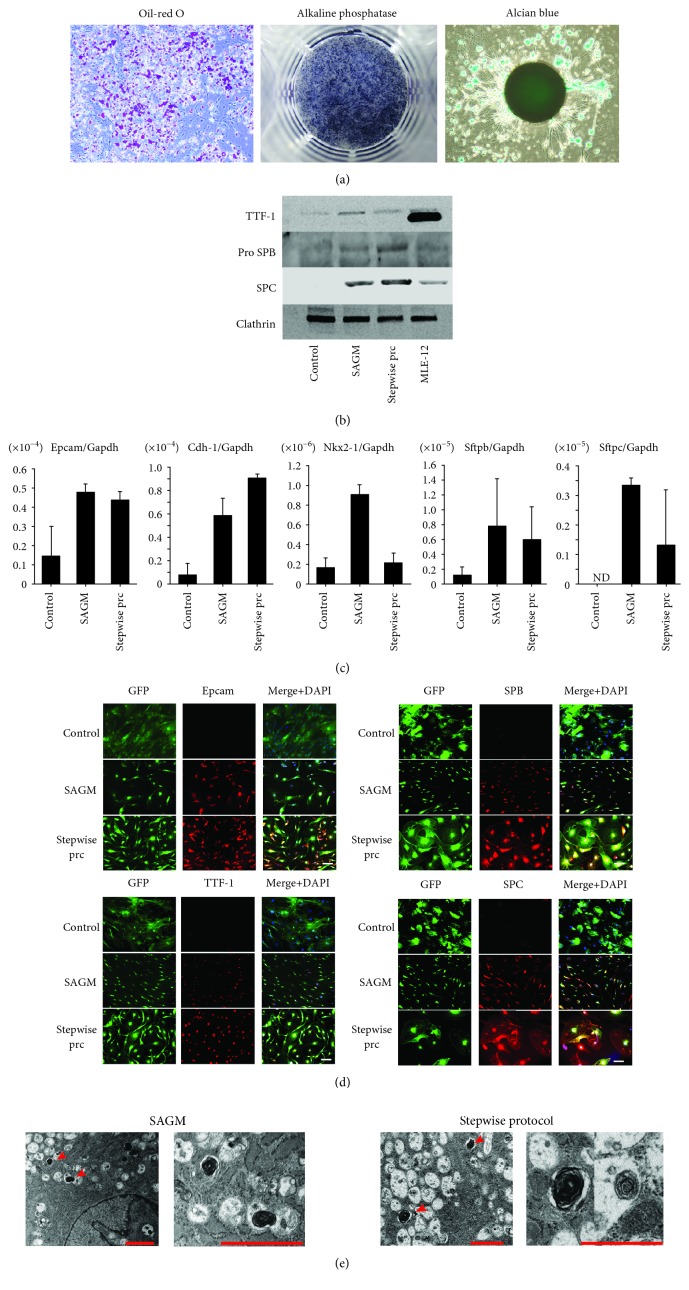
Differentiation into trilineage cells and alveolar epithelial cells of adipose-derived stem cells (ADSCs). (a) Histological analysis of differentiation of ADSCs into adipogenic, osteogenic, and chondrogenic lineages. Adipogenic differentiation was assessed with oil red O staining. Osteogenic differentiation was examined using alkaline phosphatase staining. Chondrogenic differentiation of ADSCs was examined by alcian blue staining. ADSCs have the capacity to differentiate into each mesenchymal lineage. Scale bars: 50 *μ*m. (b) Western blot analysis of ADSCs. Expression of TTF-1, Pro SPB, and SPC is shown. MLE-12 is a murine lung epithelial cell line. (c) Real-time quantitative RT-PCR analysis of ADSCs. ADSCs were cultured in small airway growth medium (SAGM) or with the stepwise protocol for 28 days. Expression of EPCAM and Cdh-1 (epithelial markers) and Nkx2-1, Sftpb, and Sftpc (type 2 alveolar epithelial cell markers) was analyzed with RT-PCR on day 28 (*n* = 3). Each value was normalized to the level of Gapdh. ND: not detected. (d) Immunofluorescence staining of ADSCs with anti-EPCAM, anti-TTF-1, anti-SPB, and anti-SPC. Control: ADSCs cultured in growth medium; SAGM: ADSCs cultured in SAGM; stepwise prc: ADSCs cultured with the stepwise protocol. Scale bars: 100 *μ*m. (e) Transmission electron microscopy of ADSCs. Lamellar body-like structures were observed in ADSCs cultured in SAGM or with the stepwise protocol for 28 days. Scale bars: 2 *μ*m.

The multipotential ability to differentiate into fat, cartilage, and bone is one of the classical definitions of MSCs and is the most important feature. Therefore, we analyzed the multilineage differentiation potential of ADSCs, including differentiation into adipogenic, osteogenic, and chondrogenic cells. ADSCs were isolated from the subcutaneous fat of mice and cultured to induce differentiation into adipocytes, osteocytes, and chondrocytes in induction medium for fat, bone, and cartilage, respectively. Positive staining with oil red O, alkaline phosphatase, and alcian blue indicated that ADSCs could differentiate into adipocytes, osteocytes, and chondrocytes, respectively (Figure 1(a)). Thus, ADSCs have the potential for multilineage differentiation.

### 3.2. In Vitro Differentiation of ADSCs into Alveolar Epithelial Cells

We investigated whether ADSCs can differentiate into epithelial cells, especially type 2 alveolar epithelial cells. ADSCs were cultured in two types of differentiation conditions, small airway growth medium (SAGM) and a stepwise differentiation protocol based on a previous study [33]. We analyzed epithelial and type 2 alveolar epithelial markers after ADSCs were cultured in SAGM or the stepwise protocol for 28 days. Western blot analysis findings revealed expression of alveolar epithelial markers in differentiated ADSCs (Figure 1(b)). TTF-1 expression was lower in differentiated ADSCs as compared to the MLE-12 mouse lung epithelial cell line, while the same level of Pro SPB and SPC expressions was seen in differentiated ADSCs as in MLE-12 cells. RT-PCR showed that the expression of epithelial cell markers such as epithelial cell adhesion molecule (EPCAM) and type 2 alveolar epithelial cell markers, including NK2 homeobox 1 (Nkx2-1), surfactant protein B (Sftpb), and surfactant protein C (Sftpc) was higher in ADSCs after inducing differentiation, as compared with ADSCs cultured in growth medium (Figure 1(c)). In addition, following induction to differentiation, immunocytochemistry results showed that ADSCs were positive for EPCAM, thyroid transcription factor-1 (TTF-1), surfactant protein B (SPB), and surfactant protein C (SPC) (Figure 1(d)). Additionally, high resolution images revealed vesicle staining of SPC in the cytoplasm (Supplemental Fig.  1). Transmission electron microscope ultrastructural analysis showed characteristic lamellar body-like inclusions, which are secretory organelles specific to alveolar type 2 epithelial cells (Figure 1(e)). These data demonstrated that ADSCs differentiated into alveolar epithelial cells, particularly alveolar type 2 cells.

### 3.3. ADSCs Engrafted in Emphysematous Lungs of Mice

**Figure 2:**
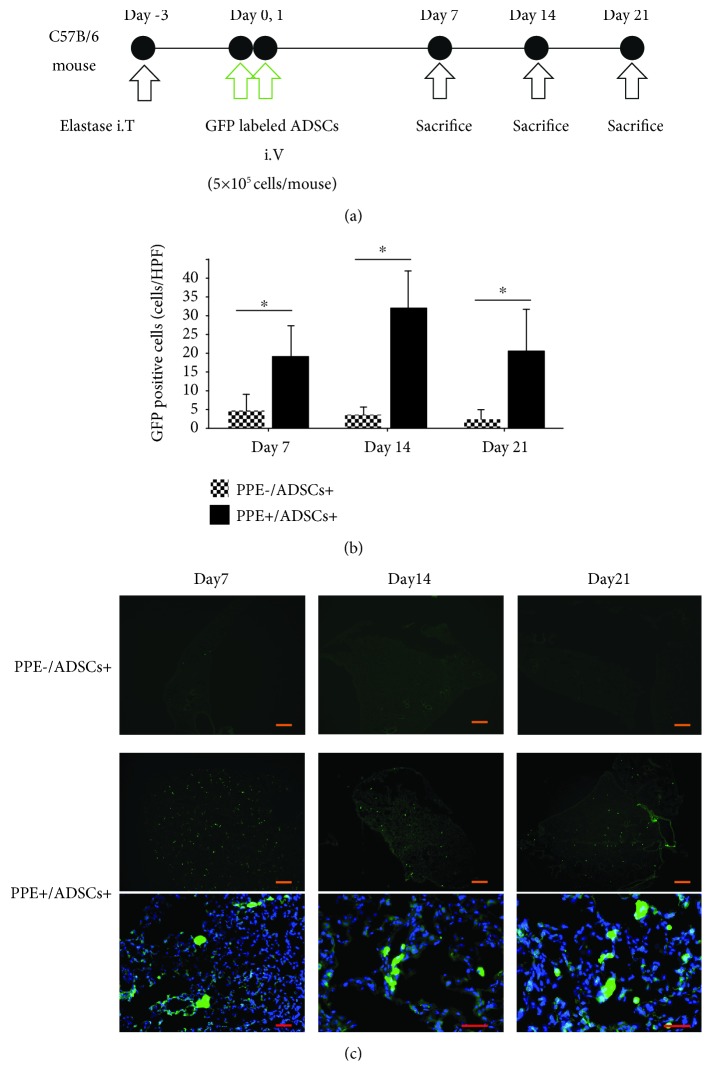
Homing and engraftment of ADSCs in the lung of emphysematous model mice. (a) Protocol of emphysema induction in model mice. Emphysema was induced in C57BL/6 mice by intratracheal administration of elastase (porcine pancreatic elastase (PPE), 1.5 IU/mouse). After 3 days, ADSCs were intravenously administered (5 × 10^5^ cells/mouse) at days 0 and 1. Histological analysis was conducted on days 7, 14, and 21. (b) The number of ADSCs was counted on day 7, day 14, and day 21 after administration of ADSC to the PPE- group and PPE+ group to compare the GFP+ engraftment in the lung (*n* = 3). ^∗^ *P* < 0.05. Scale bars: 500 *μ*m. HPF: high-power fields, ×20. (c) Representative images of GFP-labeled ADSCs in the lung of the PPE- group and PPE+ group on days 7, 14, and 21 side by side. Scale bars in low power fields are 500 *μ*m and in high-power fields are 50 *μ*m.

To investigate the engraftment capacity of ADSCs into the emphysematous lungs, we randomly divided mice into the following two groups: porcine pancreatic elastase (PPE)−/ADSCs+ mice, which were given 50 *μ*l PBS intratracheally and ADSCs intravenously, and PPE+/ADSCs+ mice, which were given PPE intratracheally to induce emphysema and ADSCs intravenously. We observed engraftment in histological findings obtained at 7, 14, and 21 days after administration of ADSCs (Figure 2(a)). Immunohistochemical analysis of mice in the PPE+/ADSCs+ group showed the presence of green fluorescent protein- (GFP-) positive cells at 7, 14, and 21 days after transplantation, whereas few GFP-positive cells were detected in the PPE−/ADSCs+ group after transplantation at all time points. Furthermore, we randomly selected 10 different areas from each sample and determined the number of cells with GFP positivity, which revealed that the GFP-positive cell count was significantly higher in the PPE+/ADSCs+ group than in the PPE−/ADSCs+ group on each examined day after administration (*P* < 0.05) (Figure 2(b)). We detected GFP-labeled ADSCs even at day 21 (Figure 2(c)). These data demonstrated that ADSCs could be engrafted into the injured tissue.

### 3.4. ADSCs Provide New Alveolar Epithelial Cells via Differentiation in the Emphysematous Lung

**Figure 3:**
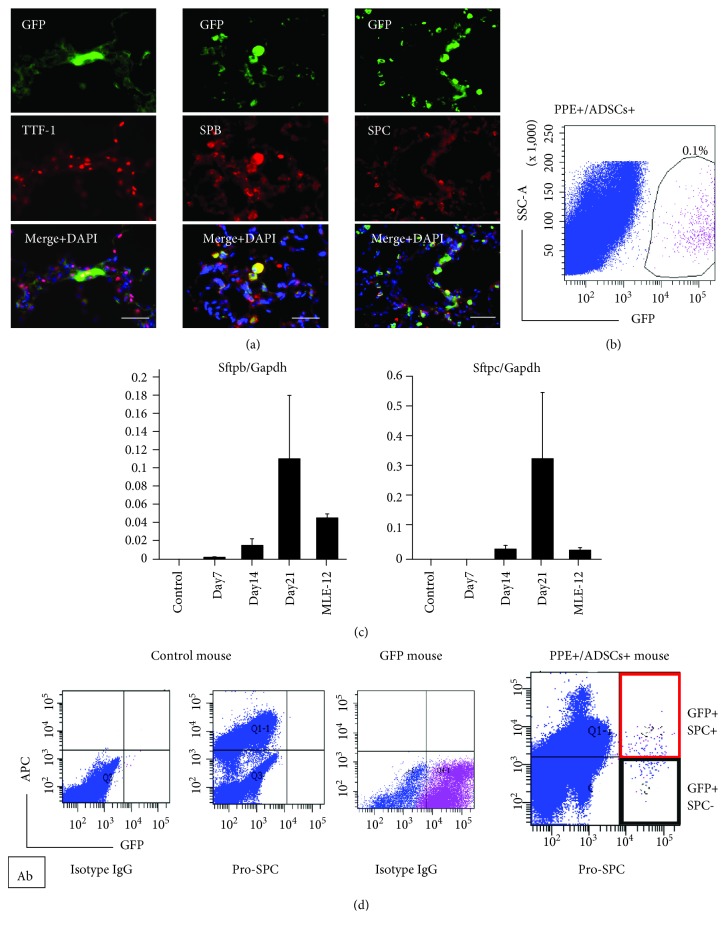
Differentiation of engrafted GFP-labeled ADSCs in the lung of the emphysematous mouse model. (a) Immunohistochemical staining of anti-TTF-1, anti-SPB, and anti-SPC in GFP-positive cells in an emphysematous mouse lung. Scale bars: 50 *μ*m. (b) Isolation of GFP-labeled ADSCs with fluorescence-activated cell sorting (FACS) on day 21. (c) Real-time quantitative RT-PCR analyses of Sftpb and Sftpc in GFP-labeled ADSCs sorted from a murine lung with FACS (each day, *n* = 6). (d) Cells were isolated from the lungs of control mice, GFP mice, and PPE+/ADSCs+ mice and analyzed for the percent of GFP+/SPC+ cells by flow cytometry. A representative FACS dot plot is shown.

We performed immunohistochemistry and fluorescence-activated cell sorting (FACS) to confirm that GFP-ADSCs had the ability to differentiate into alveolar epithelial cells after accumulation in the emphysematous lungs. GFP-labeled ADSCs were intravenously administered to elastase-induced emphysema lung model mice, and their lungs were analyzed with immunohistochemistry and FACS at 7, 14, and 21 days after administration of ADSCs. Immunohistochemistry showed that GFP-positive ADSCs accumulated in the emphysematous lungs, and some of the engrafted ADSCs were positive for an alveolar epithelial marker, such as TTF-1, SPB, and SPC at day 7 (Figure 3(a)). In addition, higher resolution images of immunofluorescence staining of SPC in vivo show a vesicular staining pattern for engrafted ADSCs that differentiated into type 2 alveolar epithelial cells. (Supplemental Fig.  2). FACS analysis revealed that 0.1% of total lung cells were GFP-positive in the emphysematous lungs (Figure 3(b)). Then, we sorted GFP-positive cells and confirmed the gene expression of type 2 alveolar epithelial cell markers. RT-PCR of GFP-labeled ADSCs sorted from the murine lung with FACS revealed the upregulation of Sftpb and Sftpc expression following intravenous administration to the mice (Figure 3(c)). As compared to MLE-12 cells, used as a positive control, those expression levels were higher in the GFP-labeled ADSCs. Next, to determine the proportion of cells that differentiated into type 2 alveolar epithelial cells from accumulated ADSCs, we performed FACS with GFP-labeled ADSCs and SPC labeling at day 21. The GFP-positive and SPC-positive cells are cells that differentiated into type 2 alveolar epithelial cells among all ADSCs administered into the mouse lung. We found that of all GFP-positive cells, 46.3 ± 10.6% were positive for both GFP and SPC (Figure 3(d)).

### 3.5. ADSCs Attenuate Emphysematous Changes in Mice

**Figure 4:**
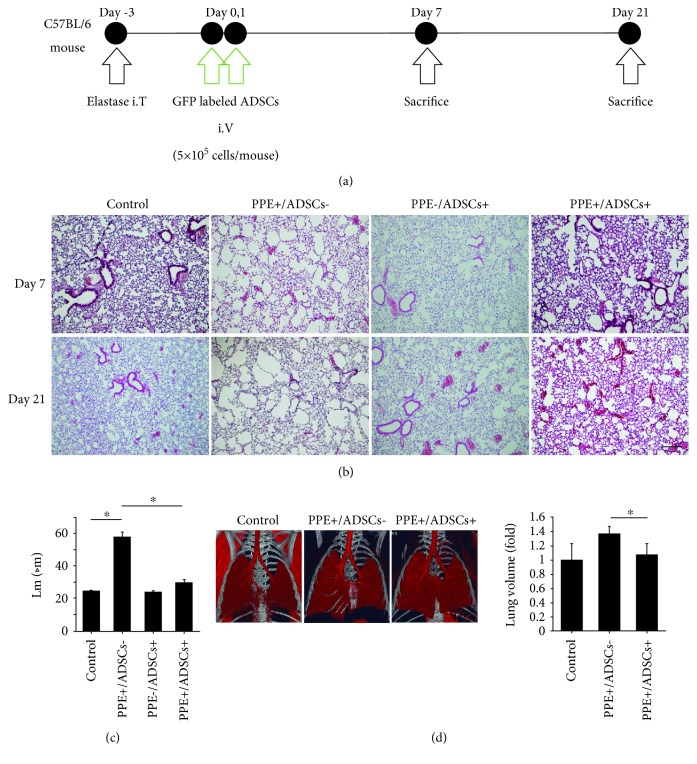
Histological effects of the administration of ADSCs in emphysema model mice. (a) The timeline of experiments. Emphysema was induced in C57BL/6 mice by intratracheal administration of elastase (porcine pancreatic elastase (PPE), 1.5 IU/mouse). After 3 days, ADSCs were intravenously administered (5 × 10^5^ cells/mouse) on days 0 and 1. Their effects were evaluated histologically on days 7 and 21. (b) Representative histological images on days 7 and 21 with hematoxylin and eosin staining. ADSCs ameliorated emphysematous changes. Scale bars, 200 *μ*m. (c) The mean linear intercepts (Lm) on day 21. Airspace enlargement was quantified by measuring the Lm (n = 3). ^∗^ *P* < 0.05. (d) Representative three-dimensional CT images. The lung volumes were measured using the product of the maximum length in the craniocaudal and lateral axes on day 21 on three-dimensional CT images (*n* = 4 in the control group, PPE+/ADSC− group, and PPE+/ADSC+ group on each day).

We divided the mice into the following four groups: (a) control, (b) PPE+/ADSCs−, (c) PPE−/ADSCs+, and (d) PPE+/ADSCs+; we then evaluated the histological and functional effects 7 and 21 days after administration of ADSCs (Figure 4(a)). Histological evaluation demonstrated obvious air space enlargement and destruction of the alveolar wall in emphysema model mice. These emphysematous changes were ameliorated by 7 and 21 days after administration of ADSCs. We show representative histopathological images in Figure 4(b). Quantitation of emphysematous changes using the mean linear intercept (Lm) method showed a strong change in the PPE+/ADSCs− group (Lm: 57.8 ± 2.7 *μ*m) compared to the control group (Lm: 24.6 ± 0.3 *μ*m), whereas the PPE+/ADSCs+ group (Lm: 29.8 ± 1.6 *μ*m) showed significant improvement (*P* < 0.05) (Figure 4(c)). Using the three-dimensional images of chest computed tomography (CT), we evaluated the volume of the lung using the product of the maximum length of the craniocaudal and lateral axes on day 21. The PPE+/ADSCs− group showed overexpansion of the lung compared with the control group. In the PPE+/ADSCs+ group, lung overexpansion was ameliorated (Figure 4(d)).

### 3.6. ADSCs Improve Lung Function in Emphysema Model Mice

**Figure 5:**
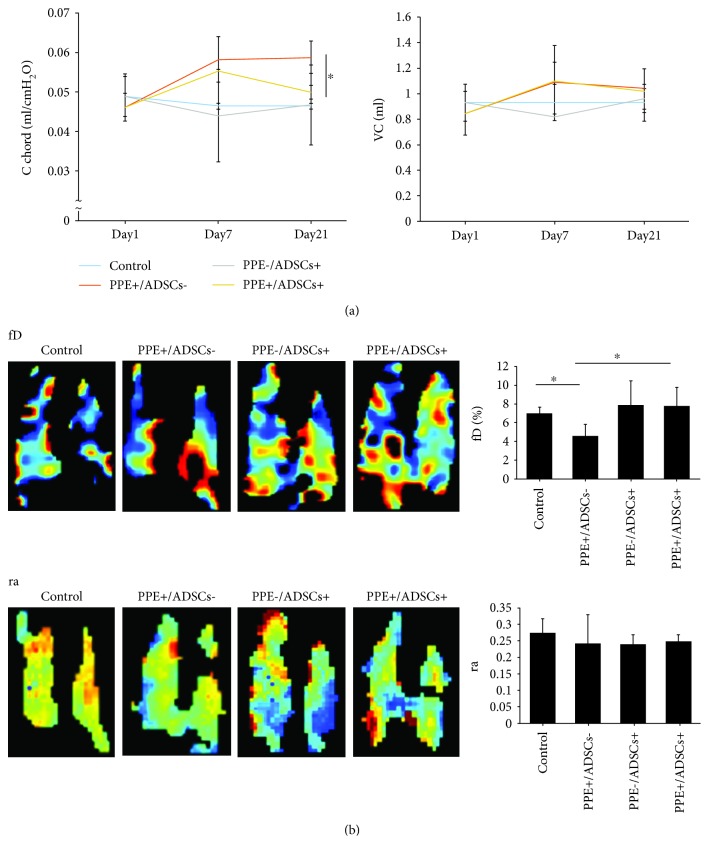
Functional effects of the administration of ADSCs in emphysema model mice. (a) A pulmonary function test was performed in mice in the following four groups: control, administered 50 *μ*l PBS intratracheally; PPE+/ADSCs−, administered PPE intratracheally; PPE−/ADSCs+, administered 50 *μ*l PBS intratracheally and injected with ADSCs intravenously; and PPE+/ADSCs+, administered PPE intratracheally and injected with ADSCs intravenously. Lung compliance indicated as C chord and vital capacity were measured by the lung function analysis system (*n* = 4 in the control group, *n* = 5 in the PPE+/ADSC− group, *n* = 6 in the PPE−/ADSC+ group, and *n* = 5 in the PPE+/ADSC+ group on each day). ^∗^ *P* < 0.01. (b) Representative hyperpolarized ^129^Xe magnetic resonance imaging (MRI). A representative map of the gas exchange efficiency (*f*_*D*_) and the ventilation efficiency (*s*) is shown. The values of *f*_*D*_ and ra were measured (*n* = 5 in the control group, *n* = 5 in the PPE+/ADSC− group, *n* = 3 in the PPE−/ADSC+ group, and *n* = 5 in the PPE+/ADSC+ group). ^∗^ *P* < 0.05.

To investigate the functional effect of the administration of ADSCs, we performed a pulmonary function test and hyperpolarized ^129^Xe magnetic resonance imaging (MRI). The pulmonary function test showed no difference in vital capacity among the groups. C chord, which indicates airway compliance, was significantly higher in the PPE+/ADSCs− group (0.06 ± 0.004 ml/cmH_2_O) than the control, whereas the PPE+/ADSCs+ group showed improvement (0.05 ± 0.002 ml/cmH_2_O; *P* < 0.01; Figure 5(a)). These data showed that ADSCs attenuated airway compliance in the emphysematous lungs of model mice. Next, we performed hyperpolarized ^129^Xe MRI to evaluate ventilation efficiency (*r*_*a*_) and gas exchange efficiency (*f*_*D*_) which indicate pulmonary function. Although there are no significant differences among *r*_*a*_ of all groups, *f*_*D*_ was decreased in the PPE+/ADSCs− group (4.6 ± 1.0%) and was improved in the PPE+/ADSCs+ group (7.8 ± 1.6%; *P* < 0.05) (Figure 5(b)). These results indicated that ADSCs improved gas exchange efficiency.

## 4. Discussion

In this study, we showed that ADSCs differentiate into type 2 alveolar epithelial cells in vitro. Furthermore, we revealed that ADSCs engrafted long term in the lungs of emphysema model mice differentiated into type 2 alveolar epithelial cells. We also showed with spirometry and ^129^Xe MRI that ADSCs functionally improved elastase-induced emphysema in the mouse model.

Our in vitro study showed that ADSCs have a multipotential ability to differentiate into various tissues including type 2 alveolar epithelial cells. Although previous studies reported that many types of MSCs can differentiate into type 2 alveolar epithelial cells, whether ADSCs can differentiate into type 2 alveolar epithelial cells in vitro has not been reported. ADSCs differentiate into ectodermal lineage cells such as neurons [34] and keratinocytes [35] or endodermal lineage cells such as hepatocytes [27]. Our results are consistent with previous studies showing that ADSCs can differentiate into cells in multiple germ layers.

We induced differentiation of ADSCs into type 2 alveolar epithelial cells with two methods, SAGM and a stepwise protocol. Several previous studies reported that BM-MSCs differentiate into type 2 alveolar epithelial cells using SAGM or modified SAGM [9, 26]. Ma et al. showed that BM-MSCs induced differentiation into type 2 alveolar epithelial cells by coculturing with lung epithelial cells (MRC-5) in modified SAGM [25]. The stepwise protocol is mainly used as a method for inducing differentiation of induced pluripotent stem (iPS) cells [33]. We confirmed the differentiation of ADSCs into type 2 alveolar epithelial cells with either SAGM or the stepwise protocol. This result strongly suggests that ADSCs can differentiate into type 2 alveolar epithelial cells. However, the cell morphology and expression of cell markers of ADSCs differentiated with the two methods were different, and investigation of the optimal differentiation induction method in the future is necessary. In addition, although the present stepwise protocol for the differentiation of iPS to alveolar type II cells seemed to be effective to induce and enhance Sftpb and Sftpc expression in ADSCs, additional studies will be necessary to apply an adequate stepwise protocol for ADSC differentiation.

Our previous studies demonstrated that HGF stimulates proliferation of respiratory epithelial cells [36], and ADSCs, via paracrine effects, improve an experimental rat model of pulmonary emphysema [17]. Many studies have discussed whether paracrine effects or engraftment and differentiation are the mechanism of action of MSCs in COPD models. However, the effects of MSCs are now thought to be paracrine and to involve immune regulation [20, 21]. This is because very few MSCs remain in the lung 1 day after systemic administration [37]. On the other hand, we found that a greater number of ADSCs accumulated in the emphysematous lungs compared to the normal lung, and these cells could be detected even on day 21. Similar to our results, Schweitzer et al. [38] also demonstrated that systemically administered ADSCs were present on day 1, and detectable ADSCs decreased, but ADSCs were detectable in the parenchyma and airway of the lungs even up to 21 days after injection. ADSCs accumulate in disordered tissues after transplantation [6, 39]. Chemokines have attracted the most attention as a factor controlling MSC homing. Several studies have shown that the interaction between stroma-derived factor-1 and its receptor, CXCR4, is one of the most important factors for homing to injured tissues [40]. However, many studies are considered controversial regarding how engrafted ADSCs function in vivo.

Our immunostaining results showed that administered ADSCs had become engrafted into the emphysema lung even on day 21 and some of the engrafted ADSCs were positive for alveolar epithelial markers such as TTF-1, SPB, and SPC. In addition, we administered ADSCs to pulmonary emphysema model mice, and GFP-labeled ADSCs in the lungs were collected by FACS. We observed that Sftpc and Sftpb increased over time in sorted ADSCs, suggesting that ADSCs differentiate into type 2 alveolar epithelial cells in vivo. These data revealed that ADSCs can differentiate into type 2 alveolar epithelial cells in injured tissue and integrate into the damaged structures. However, FACS-sorted cells from the lung may have type 2 alveolar epithelial cells contaminating them, which results in a positive result in RT-PCR, so further studies are needed. Whether MSCs have an ability to differentiate into alveolar epithelial cells remains controversial. In a study that utilized decellularized lung scaffolds, Mendez et al. reported in vitro findings showing that human MSCs differentiated into epithelial cells with stratified bodies [41]. Those results suggested that administered ADSCs may differentiate into alveolar epithelial cells in the lung tissue, which was supported by the present study. Kim et al. also found that human umbilical cord blood-derived MSCs (hCB-MSCs) induced time-dependent molecular changes in lung tissues [42]. In that study, genetic analysis results revealed that immune response regulation and oxidative stress were observed at an early stage after hCB-MSC injection and that angiogenesis and cell growth regulation became predominant and advantageous at a later stage (day 14). Our present findings are consistent with their results. Our data revealed a novel function for ADSCs in promoting repair of the damaged lung by direct differentiation into pulmonary epithelial cells. Several studies revealed that freshly isolated, primary MSCs show long-term, efficient engraftment and that high passage numbers or frozen MSCs have a reduced ability to home and differentiate [31, 43–46]. In this study, we used fresh ADSCs up to passage 3, and we detected ADSCs that had engrafted into the emphysematous lung, even on day 21, and ADSCs that had differentiated into type 2 alveolar epithelial cells in vivo. However, we still need to analyze the engraftment of ADSCs over a longer time.

As in previous reports, ADSCs led to histological improvement in pulmonary emphysema. In addition, we found that ADSCs functionally improved elastase-induced emphysema. MSCs have been widely applied in preclinical studies of COPD models, among which exogenous MSCs have been shown to repair the structure and improve the function of the injured respiratory system in COPD models [47]. We performed a pulmonary function test similar to clinical lung volume measurement (spirometry) in the emphysematous mouse model. The present results showed that ADSCs improve airway compliance in the emphysematous lungs, whereas no significant difference was found in regard to lung vital capacity (VC). This is considered to be due to the fact that VC is susceptible to various factors such as body weight, since VC of mice is small, as previously reported by De Vleeschauwer et al. [48]. In addition, we consider that the significant difference in chord compliance (C chord) for the analysis of lung function in COPD mice is important. Regarding airway compliance, C chord [46] and gas exchange efficiency are important factors in evaluating lung function, but no studies have evaluated these functions. We evaluated the effect of ADSCs on gas exchange efficiency (*f*_*D*_) using ^129^Xe MRI in this study for the first time. ^129^Xe MRI analyzes lung function in a way that cannot be done by CT or histological evaluation. ^129^Xe MRI is commonly used in preclinical studies involving small rodents with various pulmonary diseases, because ^129^Xe MRI for pulmonary functional imaging makes it possible to simultaneously evaluate fractional ventilation (*r*_*a*_) and *f*_*D*_ in the same animals, as previously described by Imai et al. [49]. Changes in *f*_*D*_ with ^129^Xe MRI can be useful for the diagnosis and evaluation of early stages of COPD [31]. We found that ADSC administration improved not *r*_*a*_ but *f*_*D*_, indicating that ADSCs can attenuate a ventilation-perfusion mismatch, probably due to increased perfusion in the lungs. We also previously reported that their administration enhanced epithelial cell proliferation and promoted angiogenesis in pulmonary vasculature, leading to the restoration of pulmonary function affected by emphysema [17], which was confirmed by the present findings.

The current main therapies for COPD are bronchodilators, which reduce the symptoms but cannot suppress the decline in respiratory function. Due to the limitations of these current COPD treatments, we are interested in the development of new regeneration therapies that improve respiratory function by repairing damaged alveolar structures. Many preclinical studies have reported that MSCs improve the emphysematous mouse model. Although administration of MSCs has been shown to be safe in patients with COPD, clinical trials in this field have not demonstrated strong therapeutic effects, such as pulmonary function improvement or mortality reduction [50–52]. Therefore, new advanced methods may be needed to increase MSC potency, such as increasing the differentiation efficiency and paracrine effects and achieving more persistent engraftment. Cho et al. reported that spheroid MSCs maintain stem cellularity, show high anti-inflammatory action, increase growth factor secretion, and increase differentiation potential compared to MSCs cultured in two dimensions [19]. Hong et al. reported that ADSCs pretreated with the peroxisome proliferator-activated receptor-*γ* agonist, pioglitazone, have more potent therapeutic effects than non-pre-treated ADSCs for the repair of alveolar destruction in emphysema mouse models [53]. Also, several papers have shown that fresh but not frozen MSCs and MSCs from younger donors are more effective [43, 54]. The conditions of this study can also be improved concerning the route of administration, cell type, number of injected cells, and frequency or timing of administration of ADSCs for a more effective use for COPD treatment.

Our study has a number of limitations. First, our experiments were performed in a preclinical rodent model, and we need to consider the clinical relevance. Second, we did not analyze the function of alveolar epithelial cells derived from ADSCs in vivo. We should further examine the balance between paracrine effects and differentiation of ADSCs. Another limitation is that the mechanisms by which ADSCs differentiate into alveolar epithelial cells in vivo have not been elucidated. In addition, we did not examine the side effects due to the administration of ADSCs. Long-term side effects should be considered.

## 5. Conclusion

We report that ADSCs can differentiate into alveolar epithelial cells. ADSCs accumulated in the emphysematous lung and differentiated into type 2 alveolar epithelial cells in vivo. Furthermore, we revealed that ADSCs improved pulmonary emphysema model mice, not only histologically but also functionally. ADSCs have therapeutic potential as a source of alveolar epithelial cells for treating pulmonary emphysema. These data may be important for exploring the efficacy of ADSCs in a clinical study of COPD.

## Data Availability

The data used to support the findings of this study are included within the article.

## Supplementary Materials

Supplementary Materials Supplementary 1. Supplemental Experimental Procedures: the materials and methods for the differentiation of ADSCs into adipocytes, osteocytes, chondrocytes, and alveolar epithelial cells in vitro. Supplementary 2. Figure S1: related to Figure 1. Higher resolution images for the immunofluorescence staining of ADSCs with anti-SPC. Supplementary 3. Figure S2: higher resolution images for the immunohistochemical staining of anti-SPC in GFP-positive cells in an emphysematous mouse lung.
