# Supplementary material for: Adipose Tissue-Derived Stem Cells Have the Ability to Differentiate into Alveolar Epithelial Cells and Ameliorate Lung Injury Caused by Elastase-Induced Emphysema in Mice
Source: Stem Cells Int. 2019 Jun 10;2019:5179172. doi: 10.1155/2019/5179172 (PMC6590553; doi:10.1155/2019/5179172)
Supplement: Supplementary Materials — Supplementary 1. Supplemental Experimental Procedures: the materials and methods for the differentiation of ADSCs into adipocytes, osteocytes, chondrocytes, and alveolar epithelial cells in vitro. Supplementary 2. Figure S1: related to Figure 1. Higher resolution images for the immunofluorescence staining of ADSCs with anti-SPC. Supplementary 3. Figure S2: higher resolution images for the immunohistochemical staining of anti-SPC in GFP-positive cells in an emphysematous mouse lung. [file 5179172.f1.pdf]

Supplementary 1.

SUPPLEMENTAL EXPERIMENTAL PROCEDURES

Adipogenic differentiation

We seeded ADSCs at a density of 1 × $10^5/cm^2$ and cultured the cells in 10% FBS-DMEM until confluent. After reaching confluence, we induced cells to differentiate into adipocytes with adipogenic differentiation medium (DMEM containing 10% FBS, 1% Penicillin-Streptomycin-Amphotericin B Suspension, 0.87 μM insulin, 0.25 μM dexamethasone, 0.5 mM methyl-isobutyl-xanthine, and 5 μM pioglitazone (all from Sigma-Aldrich)) for 14 days [1]. To observe lipid accumulation, we stained cells with oil red O stain. Cells were fixed with 10% formalin for 10 min, washed, and stained with oil red O solution (0.5% oil red O (Sigma-Aldrich) in 60% isopropanol (FUJIFILM Wako Pure Chemical Corporation, Osaka, Japan)) for 15 min. We eluted the stained dye with 60% isopropanol.

Chondrogenic differentiation

We dropped the micromass of ADSCs (10 μl of 1.0 × 10^8 cells/ml suspension) onto the center of the well of 24-well tissue culture plates. After 3 h at 37°C, we added chondrogenic differentiation medium (high glucose-DMEM (Sigma-Aldrich), 1% ITS Premix® (Corning

Incorporated, Corning, NY), 50 μg/ml ascorbic acid-2-phosphate (Sigma-Aldrich), and 10 ng/ml transforming growth factor β-3 (Peprotech, Rocky Hill, NJ)) and cultured the cells at 37°C in humidified 5% CO_2 [2]. After 7 days, cells were fixed with 4% paraformaldehyde (Wako) at room temperature for 5 min and washed with PBS and 0.1 N HCl for 3 min. We stained the cells in 1% alcian blue solution (Sigma-Aldrich).

Osteogenic differentiation

ADSCs were seeded at 2×10^4 cells/cm^2 in growth medium. The next day, we replaced the medium with osteogenesis differentiation medium (DMEM, 10% FBS, 50 μg/ml ascorbic acid-2-phosphate, 20 mM α-glycerophosphate (all from Sigma-Aldrich)) [3]. On day 7, we confirmed differentiation by assessing alkaline phosphate activity. The cells were washed with PBS, fixed with 4% paraformaldehyde for 5 min at room temperature, and rinsed with distilled water. We added enough BCIP/NBT substrate solution (PromoCell GmbH, Heidelberg, Germany) to cover the cellular monolayer. We incubated the cells at room temperature in the dark for 30 min, and then washed the cells with distilled water.

Alveolar epithelial differentiation

To investigate whether type 2 alveolar epithelial cells can differentiate from ADSCs,

differentiation induction was carried out with two conditions. The first condition involved SAGM (Lonza, Basel, Switzerland), which is a peripheral airway growth medium, and the other involved a stepwise protocol in which differentiation was induced according to the developmental process of the lungs [4]. We cultured ADSCs with growth medium until the cells were confluent, and then replaced the medium with SAGM and cultured the cells for 28 days. In the stepwise protocol, we incubated the ADSCs in differentiation medium containing 0.2% FBS, 10 ng/ml Activin A (R&D Systems, Minneapolis, MN), and 10 ng/ml Wint3a (R&D Systems) for 4 days after the cells reached confluence. For step 2, the medium was changed to DMEM supplemented with 0.2% FBS, 50 ng/ml Noggin (R&D Systems), and 5 μM SB431542 (Tocris Bioscience, Bristol, UK) for 2 days. For step 3, we cultured the cells with DMEM containing 0.2% FBS, 20 ng/ml Wint3a, 2.5 ng/ml keratinocyte growth factor (KGF), 5 ng/ml FGF-10, and 5 ng/ml epidermal growth factor (R&D Systems) for 7 days. For step 4, the medium was changed to DMEM containing 0.2% FBS, 20 ng/ml Wint3a, 2.5 ng/ml KGF (R&D Systems), and 5 ng/ml FGF-10 (R&D Systems) for 7 days. For step 5, the ADSCs were incubated with SAGM for 7 days. From step 1 to step 4, we changed the medium every other day.

SUPPLEMENTAL REFERENCES

1 Lama VN, Smith L, Badri L, et al. Evidence for tissue-resident mesenchymal stem cells in human adult lung from studies of transplanted allografts. J Clin Invest. 2007;117:989-996.

2 Zhang LM, Su PQ, Xu CX, et al. Chondrogenic differentiation of human mesenchymal stem cells: a comparison between micromass and pellet culture systems. Biotechnol Lett. 2010;32:1339-1346.

3 Tateishi K, Ando W, Higuchi C, et al. Comparison of human serum with fetal bovine serum for expansion and differentiation of human synovial MSC: Potential feasibility for clinical applications. Cell Transplant. 2008;17:549-557.

4 Ghaedi M, Calle EA, Mendez JJ, et al. Human iPS cell-derived alveolar epithelium repopulates lung extracellular matrix. J Clin Invest. 2013;123:4950-4962.

Supplementary 2.

Supplemental Figure 1

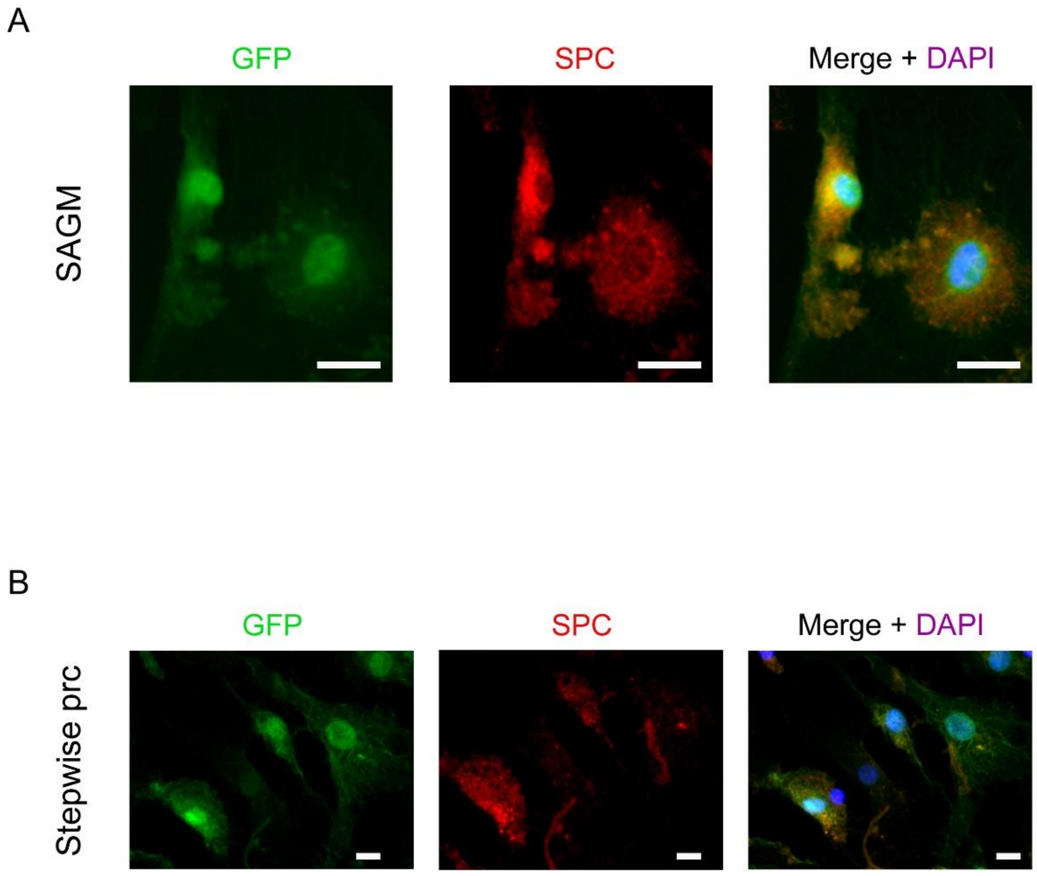

Supplemental Figure 1. Related to Figure 1.

Higher resolution images for the immunofluorescence staining of ADSCs with anti-SPC.

(A) SAGM: ADSCs cultured in SAGM. Scale bars, 20 μm.

(B) stepwise prc: ADSCs cultured with the stepwise protocol. Scale bars, 20 μm.

Supplementary 3.

Supplemental Figure 2

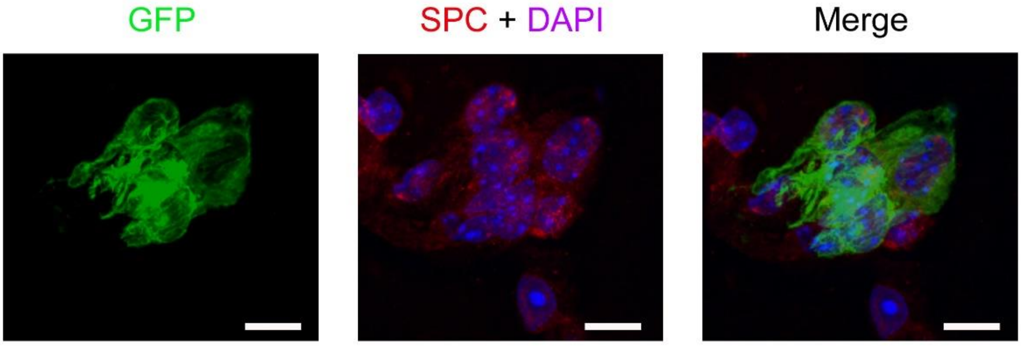

Supplemental Figure 2. Related to Figure 3A.

Higher resolution images for the Immunohistochemical staining of anti-SPC in GFP-positive cells in emphysematous mouse lung. Scale bars, 10 μm.
